# The Use of Schisandrin B to Combat Triple-Negative Breast Cancers by Inhibiting NLRP3-Induced Interleukin-1β Production

**DOI:** 10.3390/biom14010074

**Published:** 2024-01-05

**Authors:** Chun-Ming Chang, Ting-Ruei Liang, Ho Yin Pekkle Lam

**Affiliations:** 1 Department of General Surgery, Hualien Tzu Chi Hospital, Buddhist Tzu Chi Medical Foundation, Hualien 970473, Taiwan; 2 School of Medicine, Tzu Chi University, Hualien 970374, Taiwan; 3 PhD Program in Pharmacology and Toxicology, Tzu Chi University, Hualien 970374, Taiwan; 4 Department of Biochemistry, School of Medicine, Tzu Chi University, Hualien 970374, Taiwan

**Keywords:** triple-negative breast cancer, Schisandrin B, recurrence, inflammasome activation

## Abstract

Triple-negative breast cancer (TNBC) is the most aggressive and fatal breast cancer subtype. Nowadays, chemotherapy remains the standard treatment of TNBC, and immunotherapy has emerged as an important alternative. However, the high rate of TNBC recurrence suggests that new treatment is desperately needed. Schisandrin B (Sch B) has recently revealed its anti-tumor effects in cancers such as cholangiocarcinoma, hepatoma, glioma, and multi-drug-resistant breast cancer. However, there is still a need to investigate using Sch B in TNBC treatment. Interleukin (IL)-1β, an inflammatory cytokine that can be expressed and produced by the cancer cell itself, has been suggested to promote BC proliferation and progression. In the current study, we present evidence that Sch B can significantly suppress the growth, migration, and invasion of TNBC cell lines and patient-derived TNBC cells. Through inhibition of inflammasome activation, Sch B inhibits interleukin (IL)-1β production of TNBC cells, hindering its progression. This was confirmed using an NLRP3 inhibitor, OLT1177, which revealed a similar beneficial effect in combating TNBC progression. Sch B treatment also inhibits IL-1β-induced EMT expression of TNBC cells, which may contribute to the anti-tumor response.

## 1. Introduction

Breast cancer is one of the most common malignant tumors in females, with a high mortality rate and continuously increasing incidences [1]. Breast cancer can be divided into different subtypes based on the presence of its molecular markers: estrogen receptor (ER), progesterone receptor (PR), and human epidermal growth factor receptor 2 (HER2). The lack of all three markers represents triple-negative breast cancer (TNBC). TNBC, which constitutes 15–20% of all breast cancers, is the most aggressive and fatal subtype [1], with an average survival rate of approximately 10 months and a 5-year survival rate of only 65% in the case of regional tumors and 11% in the case of metastatic tumors [1]. In addition, TNBC has a high incidence of visceral metastasis and is naturally recurrent [2]; therefore, the prognosis is usually unfavorable.

In current clinical practice, surgery, chemotherapy, and radiotherapy remain the standard treatment of TNBC. The use of platinum-based agents or other targeted agents such as poly ADP ribose polymerase (PARP) inhibitors may yield higher therapeutic success [3]; however, the specific adjuvant regimens that may be effective for TNBC remain incompletely defined. While TNBC is considered a more immunogenic subtype than other breast cancer subtypes, immunotherapy has emerged as an important alternative for TNBC [4]. One important immunotherapeutic approach is anti-programmed death (PD)-1 or PD-ligand 1 (L1) agents such as atezolizumab, pembrolizumab, and durvalumab, as recent studies have demonstrated that tumor-infiltrating lymphocytes expressing a high level of PD-1 or TNBC representing high PD-L1 levels are associated with a more favorable outcome [4]. However, immunotherapy might only work for a limited number of TNBC patients, not to mention that resistance to immunotherapy has been characterized clinically [5]. Therefore, more effort must be made to find alternative therapeutics for TNBC. 

In recent years, studies have begun investigating the potential of herbal compounds in TNBC treatment [6]. Natural compounds have the advantage of being less toxic and having fewer side effects on patients. Schisandrin B (Sch B), a compound extracted from *Schisandra chinensis*, has previously been used to treat liver diseases [7,8,9]. Lately, investigations have claimed the beneficial effect of Sch B against different tumors such as cholangiocarcinoma [10], hepatoma [11], gastric cancer [12], glioma [13], prostate cancer [14], and multi-drug-resistant breast cancer [15]. Research has suggested that Sch B is able to restore the drug sensitivity of multi-drug-resistant MCF-7 cells [15]. Sch B has also been found to inhibit metastasis of 4T1 cells in mouse models [16]. A recent study using Sch B against three TNBC cell lines, MDA-MB-231, BT-549, and MDA-MB-468, proposed a STAT3-inhibitory mechanism of Sch B in killing TNBC cells [17]. However, the challenge remains in identifying a more detailed mechanism for using Sch B against TNBC. 

## 2. Materials and Methods

### 2.1. Cell Lines, Patient-Derived Cells, and Cell Culture

Two human breast cancer cell lines, MDA-MB-231 (ATCC#: HTB-26) and Hs-578T (ATCC#: HTB-126), were obtained from the Food Industry Research and Development Institute (Hsinchu, Taiwan). Cells were maintained in Dulbecco’s modified Eagle’s medium (DMEM; HyClone, Cytiva, Marlborough, MA, USA) supplemented with 10% fetal bovine serum (FBS; Gibco; Thermo Fisher Scientific, Waltham, MA, USA), 1% L-glutamine (HyClone), 1% nonessential amino acids (NEAA; HyClone), 1% sodium pyruvate (HyClone), and 1% penicillin–streptomycin (HyClone). 

One human normal breast epithelial cell line, MCF-10A (ATCC#: CRL-10317), was obtained from the American Type Culture Collection (ATCC; Manassas, VA, USA). Cells were maintained in DMEM/Nutrient Mixture F-12 (F12) medium containing 10% FBS, 1% L-glutamine, 1% NEAA, 1% sodium pyruvate, 1% penicillin–streptomycin, 10 ng/mL human epidermal growth factor (EGF; MP Biomedicals, Steven Hills, NSW, Australia), 5 mg/mL insulin (MP Biomedicals), and 5 ng/mL 17-beta-estradiol (MP Biomedicals).

One human breast cancer sample, TCHBC5, was obtained during surgery. The breast cancer specimen was put immediately into sterile tubes containing 10 mL of FBS-free Roswell Park Memorial Institute (RPMI) 1640 medium (HyClone) with 1% penicillin and streptomycin and was transferred to the laboratory in ice. The specimen was washed in FBS-free RPMI 1640 medium, followed by the removal of fat and necrotic tissues and dissection into approximately 1 × 1 mm fragments. The fragments were then incubated at 37 °C with type III collagenase (Worthington, Chicago, IL, USA; 2 mg/mL in RPMI 1640 medium) for 5 h. The cells were checked every 30 min until complete digestion. Collagenase activity was blocked by adding 10% FBS-containing RPMI 1640 medium before the cells were pelleted by centrifugation at 500× *g* for 5 min. The cell pellets were then resuspended in a DMEM/F12 medium containing the same supplements used in culturing MCF-10A cells. The cells were seeded in Petri dishes as passage 0 and maintained under standard operating procedures. Clinicopathological data were collected from the patient’s medical records and are presented in Table 1.

All cell lines were handled under standard operating procedures and incubated in a humidified atmosphere of 5% CO_2_ at 37 °C. The human study was approved by the Research and Ethical Review Committees and Internal Review Board of Hualien Tzu Chi Hospital (IRB109-189-A). Written informed consent was obtained from the patient for their tissue to be used in research.

### 2.2. Cell Proliferation Assay

Cells were plated at a density of 1 × 10^3^ cells/well in 96-well plates. After culture overnight, the cells were treated with different concentrations of Sch B (Chengdu Alfa Biotechnology, Chengdu City, China) for 24 or 48 h. The proliferation of cells was determined using Cell Counting Kit-8 (CCK-8; Cyrusbioscience, Taipei, Taiwan), according to the manufacturer’s instructions. The absorbance of the reaction was measured at a wavelength of 450 nanometers (nm). 

### 2.3. Clonogenic Assay

Cells were plated at a density of 1 × 10^5^ cells/well into 6-well plates and treated with different concentrations of Sch B. After 24 h of treatment, the cells were re-plated into a new 6-well plate with 2 × 10^4^ cells/well. The cells were cultured for 15 days with a drug-free medium. After that, the cells were washed with PBS, fixed with methanol, and stained with 1% crystal violet. 

### 2.4. Transwell Migration and Invasion Assay

A Transwell migration and invasion assay was carried out using 24-well plates with chambers of 8 µm pore polycarbonate membrane (Cat:#3422, CORNING, Corning, NY, USA). For the migration assay, 2 × 10^4^ cells/well were seeded in the upper chamber in 100 μL of serum-free medium, and 600 μL of complete medium was added to the lower chamber. After 24 h culture, the non-migrated cells at the upper surface of the membrane were removed with sterile cotton swabs, and cells at the lower surface were fixed with methanol and stained with 1% crystal violet.

A similar procedure was carried out for the invasion assay except that inserts were pre-coated with Matrigel (BD Biosciences, Franklin Lakes, NJ, USA) 6 h before cells were seeded onto the membrane. The cells were cultured for 48 h before performing the staining procedures.

### 2.5. RNA Isolation, cDNA Synthesis, and Quantitative Real-Time PCR (qRT-PCR)

Cells were seeded at a density of 8 × 10^5^ cells/well in a 6 cm diameter dish. After which, the cells were treated with indicated concentrations of Sch B, 10 µM OLT1177 (Cat#: M9376; Abmole Bioscience Inc., Houston, TX, USA), or 10 ng/mL IL-1β (Cat#: A42509, Invitrogen, Thermo Fisher Scientific, Waltham, MA, USA) for 24 h. Total RNA of the cells was extracted using TRIzol reagent (Invitrogen, Thermo Fisher Scientific), according to the manufacturer’s protocol. RNA (5 μg) was used for reverse transcription with RevertAid First Strand cDNA Synthesis Kit (Fermentas International Inc., Burlington, ON, Canada). The qRT-PCR reaction was performed by 2× qPCRBIO SyGreen Blue Mix Lo-ROX (PCR Biosystems, London, UK) using the Roche LightCycler 480 system. Amplification and detection were performed as follows: 45 cycles of denaturation at 95 °C for 15 s, 60 °C for 20 s, and extension at 72 °C for 15 s. The oligonucleotide primers used are shown in Table S1. Relative gene expression was calculated using the 2^−ΔΔCT^ method, and gene expression levels were normalized to *β-actin* control.

### 2.6. Protein Extraction and Western Blotting

Cells were seeded at a density of 8 × 10^5^ cells/well in a 6 cm diameter dish. Cells were treated with Sch B, OLT1177, or IL-1β for 48 h. After washing with PBS, proteins were extracted by RIPA lysis buffer (Thermo Fisher Scientific, Inc.). Extracted proteins were separated on 10% SDS-PAGE gels and were transferred to PVDF membranes. Membranes were blocked with 5% non-fat milk and then incubated with the primary antibodies listed in Table S2. Membranes were incubated with horseradish peroxidase (HRP)-conjugated mouse anti-IgG (Cat#: AP308P; EMD Millipore, Danvers, MA, USA) or HRP-conjugated rabbit anti-IgG (Cat#: AP307P; EMD Millipore) secondary antibodies before the development of the membranes by ECL detection reagent (EMD Millipore). Protein expressions were quantified by ImageJ (National Institutes of Health, Bethesda, MD, USA) and expressed relative to α-tubulin.

### 2.7. Enzyme-Linked Immunoassay (ELISA) for IL-1β and GSDMD Concentrations

Concentrations of IL-1β in the culture media and gasdermin D (GSDMD) in the cell lysate were measured using an ELISA kit (Cat#: 437004 for IL-1β; BioLegend, San Diego, CA, USA and Cat#: RK01517 for GSDMD; ABclonal, Woburn, MA, USA) following the kit’s manual. Briefly, 96-well ELISA plates were prepared by coating the plate with 100 μL per well capture antibody overnight at 4 °C. The capture antibody was discarded, wells were washed, and 200 μL per well ELISA/ELISASPOT diluent was added to block the well for 1 h. The wells were washed and reacted with 100 μL samples or standards for 2 h. After that, 100 μL detection antibody was added into each well and incubated for 1 h. The wells were then incubated with 100 μL Avidin-HRP enzyme for 30 min. Posterior to washing, 100 μL 3,3′, 5,5′-tetramethylbenzidine (TMB) substrate per well was added for 15 min, and at the end, 10% sulfuric acid was added to each well to terminate the reaction. The optical density of the plate was measured at 450 nm. 

### 2.8. Statistical Analysis

All statistical analyses were performed using GraphPad Prism 6.01 software (GraphPad Software Inc., San Diego, CA, USA). Data are represented as the mean ± standard deviation (S.D.). A paired samples *t*-test was used to compare the two groups. One-way analysis of variance (ANOVA) was used, followed by Tukey’s honest significant difference (HSD) test, for comparisons between groups. 

## 3. Results

### 3.1. Schisandrin B Dose-Dependently Inhibits the Growth of TNBC Cells

**Figure 1:**
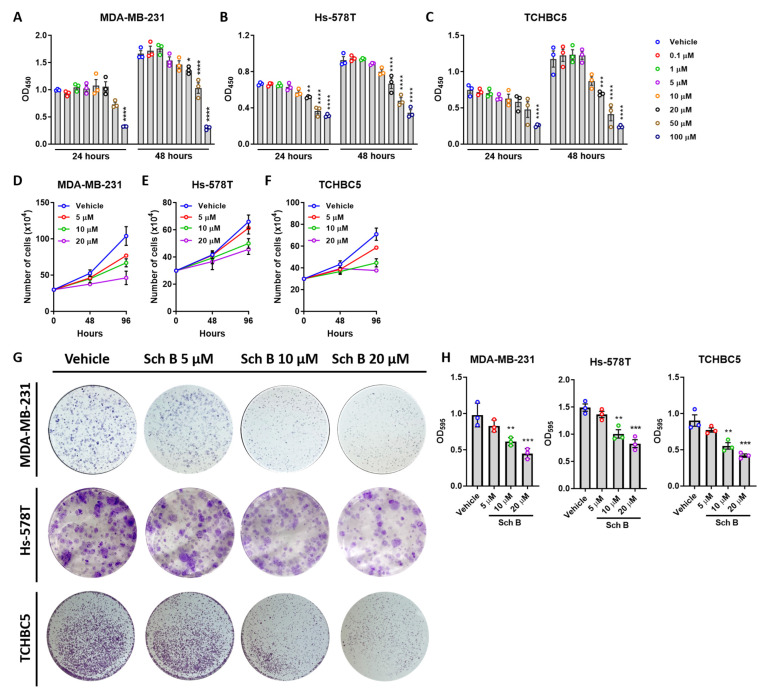
Schisandrin B (Sch B) inhibits the growth of TNBC cells. (**A**–**C**) Viability of MDA-MB-231 (**A**), Hs-578T (**B**), and TCHBC5 cells (**C**) treated with different concentrations of Sch B for 24 or 48 h. (**D**–**F**) Number of cells counted after treatment of Sch B. The cells were trypsined every 48 h, counted, and re-plated into a new 60 mm^3^ dish. (**G**,**H**) Representative images of colony formation assay of MDA-MB-231, Hs-578T, and TCHBC5 cells in a 6-well plate with surface area of 10 cm^2^ per well (**G**) and their corresponding quantification of staining intensity (**H**). Sch B dose-dependently inhibits the growth of these cells. Data points represent three independent experiments, and bar graphs are presented as mean ± SD. * *p* < 0.05, ** *p* < 0.01, *** *p* < 0.001, and **** *p* < 0.0001 compared to vehicle group. Significance according to one-way ANOVA.

To investigate the effect of Sch B on human TNBC, two TNBC cell lines, MDA-MB-231 and Hs-578T, and one clinically collected, patient-derived TNBC cell line, TCHBC5, were treated with Sch B at concentrations from 0 to 100 µM for 24 and 48 h, followed by detection of cell viability by CCK-8 assay. The results showed that at higher concentrations (starting from 5 µM for MDA-MB-231 and Hs-578T; Figure 1A,B; and 10 µM for TCHBC5; Figure 1C), Sch B decreased the viability of these TNBC cells at 48 h post-treatment in a dose-dependent manner (Figure 1A–C). As the sudden drop of cell viability at the concentration of 50 µM and 100 µM may have resulted from the cytotoxic effects of Sch B, further experiments were carried out with three Sch B concentrations: 5 µM, 10 µM, and 20 µM. To confirm the inhibitory effects of Sch B, the amount of cell growth was measured every two days, which showed similar results (Figure 1D–F). The clonogenic assay also revealed the growth-inhibitory effect of Sch B in a dose-dependent manner (Figure 1G,H). Finally, we repeated the experiment on MCF-10A cells to confirm the anti-proliferative effect is specific to cancerous cells but not normal human breast cells. Our results suggested that the viability of MCF-10A cells was not changed at 24 and 48 h by treating the cells with 0 to 20 µM Sch B (Figure S1A). Cell growth also revealed similar results, except a slight but non-significant decrease was observed in cells treated with 20 µM for 96 h (Figure S1B). Therefore, these results suggested that Sch B can provide a more prominent anti-proliferative effect on TNBC cells but not normal human breast cells. 

### 3.2. Schisandrin B Suppresses Migration and Invasion of TNBC Cells via Inhibition of Epithelial-to-Mesenchymal Transition

**Figure 2:**
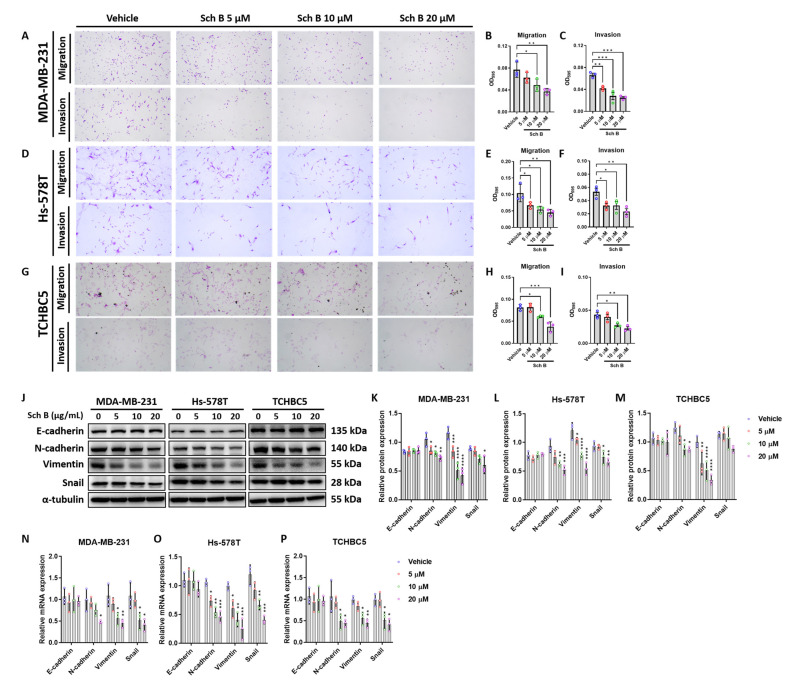
Schisandrin B (Sch B) inhibits the progression of TNBC cells by suppressing epithelial-to-mesenchymal transition (EMT). (**A**–**I**) Representative migration and invasion images and corresponding quantification of staining intensity of MDA-MB-231 (**A**–**C**), Hs-578T (**D**–**F**), and TCHBC5 cells (**G**–**I**). (**J**–**M**) Representative Western blot images of EMT markers (**J**) and expression levels of MDA-MB-231 (**K**), Hs-578T (**L**), and TCHBC5 (**M**) cells. Expression levels are relative to that of α-tubulin. Cells were treated with different concentrations of Sch B for 48 h. Original images can be found in Supplementary Materials. (**N**–**P**) mRNA expression levels of EMT markers of MDA-MB-231 (**N**), Hs-578T (**O**), and TCHBC5 (**P**) cells. Cells were treated with different concentrations of Sch B for 24 h. Protein expression level was relative to that of α-tubulin. Data points represent three independent experiments, and bar graphs are presented as mean ± SD. * *p* < 0.05, ** *p* < 0.01, *** *p* < 0.001, and **** *p* < 0.0001 compared to vehicle group. Significance according to one-way ANOVA.

Further analysis suggested Sch B can significantly impede TNBC cell migration (Figure 2A,B,D,E,G,H) and invasion (Figure 2A,C,D,F,G,I). Because epithelial-to-mesenchymal transition (EMT) plays an important role in tumor migration and invasion, certain markers of the EMT were analyzed by Western blotting (Figure 2J–M) and RT-qPCR (Figure 2N–P). Although protein expression of the epithelial marker, E-cadherin, does not change, mesenchymal markers including N-cadherin, Vimentin, and Snail were suppressed by Sch B treatment (Figure 2J–M). mRNA expression of these markers showed consistent results with their protein levels (Figure 2N–P). Altogether, these results suggested an anti-migration and anti-invasion effect of Sch B against TNBC cells.

### 3.3. Schisandrin B Inhibits NLRP3 Inflammasome Activation in TNBC Cells

**Figure 3:**
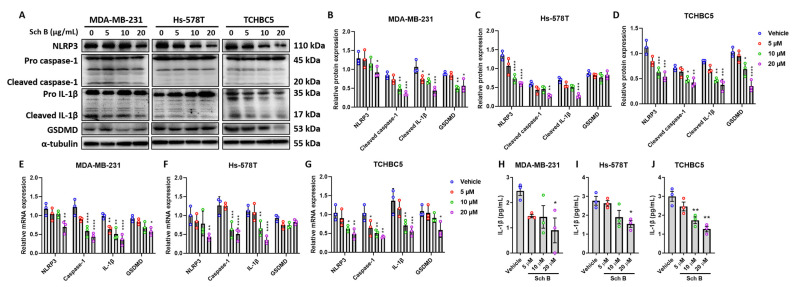
Schisandrin B (Sch B) inhibits inflammasome activation of TNBC cells. (**A**–**D**) Representative Western blot images (**A**) and protein expression levels of inflammasome markers of MDA-MB-231 (**B**), Hs-578T (**C**), and TCHBC5 (**D**) cells. Cells were treated with different concentrations of Sch B for 48 h. Original images can be found in Supplementary Materials. (**E**–**G**) mRNA expression levels of inflammasome markers of MDA-MB-231 (**E**), Hs-578T (**F**), and TCHBC5 (**G**) cells. Cells were treated with different concentrations of Sch B for 24 h. (**H**–**J**) Levels of IL-1β in the cell medium secreted by MDA-MB-231 (**H**), Hs-578T (**I**), and TCHBC5 (**J**) cells after treatment with Sch B for 24 h. Protein expression level was relative to that of α-tubulin or the pro-form of the corresponding protein. Data points represent three independent experiments, and bar graphs are presented as mean ± SD. * *p* < 0.05, ** *p* < 0.01, *** *p* < 0.001, and **** *p* < 0.0001 compared to vehicle group. Significance according to one-way ANOVA.

Inflammatory signaling operates in many cancers, contributing to the induction of EMT and tumor progression [18,19]. While studies have suggested the pivotal function of NLRP3 and IL-1β in breast cancer development [19], we next investigated whether Sch B can inhibit the activation of NLRP3 inflammasome in TNBC cells. Western blotting showed that Sch B inhibits NLRP3 expression and downstream effectors including caspase-1 and IL-1β in all tested TNBC cells in a dose-dependent manner (Figure 3A–D). However, gasdermin D (GSDMD), another inflammasome effector, was only inhibited in MDA-MB-231 and TCHBC5 cells (Figure 3A–D). This was confirmed by measuring GSDMD concentration in the cell extract, showing similar results (Figure S2A–C). mRNA levels also suggested similar effects of Sch B on TNBC cells (Figure 3E–G). In addition, decreased IL-1β production further confirmed our results (Figure 3H–J).

### 3.4. NLRP3 Inhibition Suppresses IL-1β production, Leading to Suppression of TNBC Growth and EMT Expression

**Figure 4:**
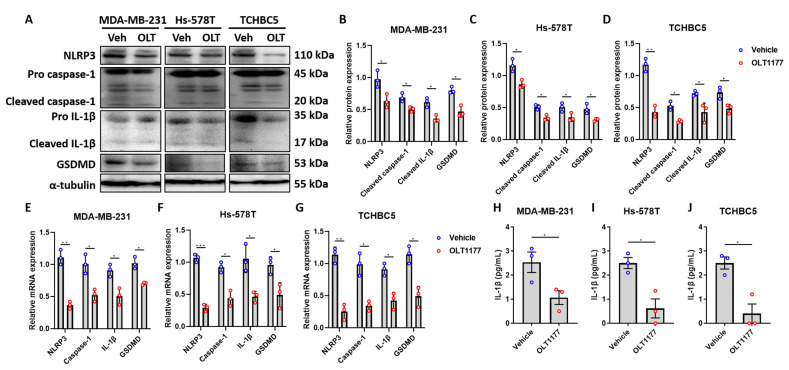
NLRP3 inhibitors suppress downstream expression of caspase-1, IL-1β, and gasdermin D in TNBC cells. (**A**–**D**) Representative Western blot images (**A**) and protein expression levels of inflammasome components of MDA-MB-231 (**B**), Hs-578T (**C**), and TCHBC5 (**D**) cells. Cells were treated with 10 µM OLT1177 for 48 h. Original images can be found in Supplementary Materials. (**E**–**G**) mRNA expression levels of inflammasome markers of MDA-MB-231 (**E**), Hs-578T (**F**), and TCHBC5 (**G**) cells. Cells were treated with different concentrations of Sch B for 24 h. (**H**–**J**) Levels of IL-1β in the cell medium secreted by MDA-MB-231 (**H**), Hs-578T (**I**), and TCHBC5 (**J**) cells. Cells were treated with 10 µM OLT1177 for 24 h. Protein expression level was relative to that of α-tubulin or the pro-form of the corresponding protein. Data points represent three independent experiments, and bar graphs are presented as mean ± SD. * *p* < 0.05, ** *p* < 0.01, and *** *p* < 0.001 compared to vehicle group. Significance according to *t*-test.

To confirm that inhibition of NLRP3 and IL-1β indeed suppresses TNBC cell growth and progression, cells were treated with an NLRP3 inhibitor, OLT1177. Our results demonstrated that inhibition of NLRP3 leads to reduced protein (Figure 4A–D) and mRNA expression (Figure 4E–G) of caspase-1, IL-1β, and GSDMD. Inhibition of NLRP3 also decreased IL-1β production in TNBC cells (Figure 4H–J). 

**Figure 5:**
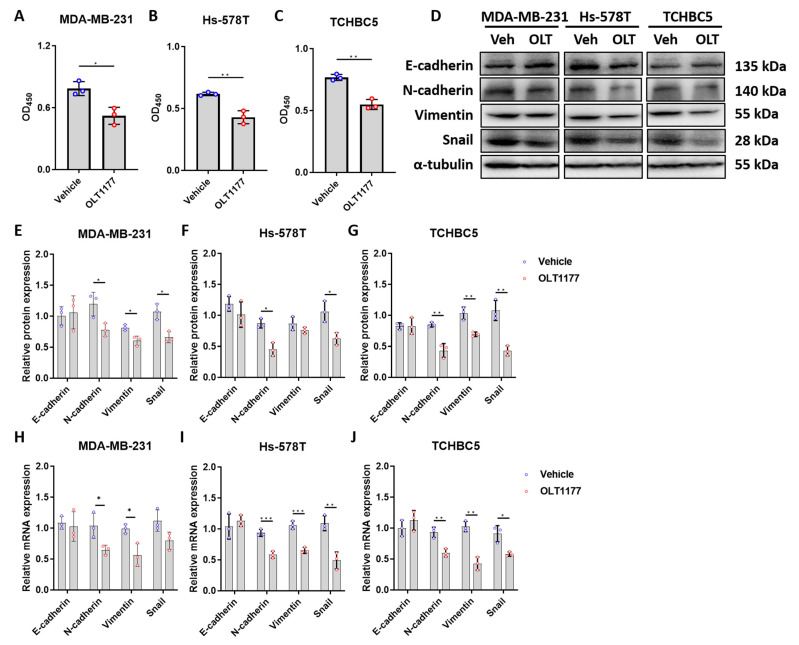
NLRP3 inhibition hinders the growth and EMT of TNBC cells. (**A**–**C**) Proliferation of MDA-MB-231 (**A**), Hs-578T cells (**B**), and TCHBC5 (**C**) cells, measured by CCK-8 assay. Cells were treated with 10 µM OLT1177 for 24 h. (**D**–**G**) Representative Western blot images (**D**) and protein expression levels of EMT markers of MDA-MB-231 (**E**), Hs-578T (**F**), and TCHBC5 (**G**) cells. Cells were treated with 10 µM OLT1177 for 48 h. Original images can be found in Supplementary Materials. (**H**–**J**) mRNA expression levels of inflammasome markers of MDA-MB-231 (**H**), Hs-578T (**I**), and TCHBC5 (**J**) cells. Cells were treated with 10 µM OLT1177 for 24 h. Protein expression level was relative to that of α-tubulin. Data points represent three independent experiments, and bar graphs are presented as mean ± SD. * *p* < 0.05, ** *p* < 0.01, and *** *p* < 0.001 compared to vehicle group. Significance according to *t*-test.

Further analysis revealed that the inhibition of NLRP3 and IL-1β further suppressed the proliferation of TNBC cells (Figure 5A–C), as well as EMT expression (Figure 5D–G for protein levels; Figure 5H–J for mRNA levels) in TNBC cells. These results confirmed the need for NLRP3 inflammasome in TNBC growth and progression. 

### 3.5. Schisandrin B Suppresses IL-1β-Induced TNBC Growth and Progression

**Figure 6:**
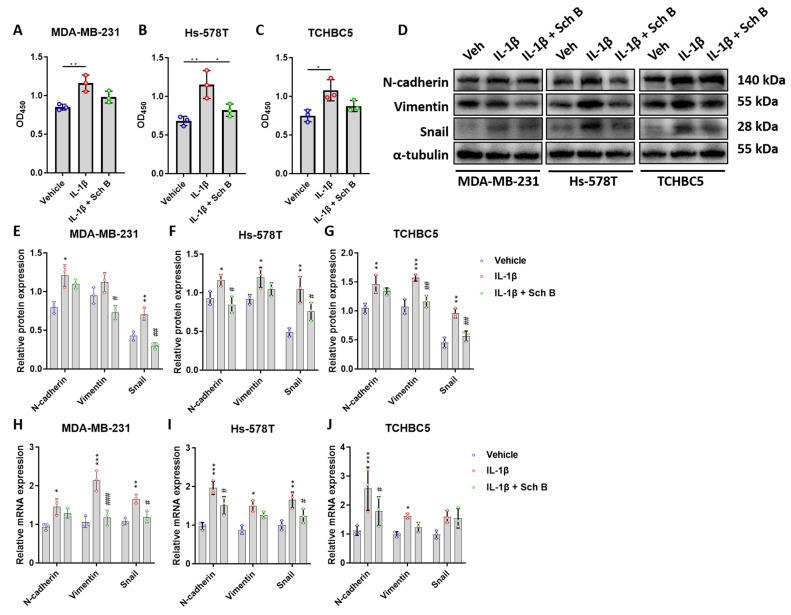
Schisandrin B (Sch B) suppresses IL-1β-induced TNBC proliferation and EMT expression. (**A**–**C**) Proliferation of MDA-MB-231 (**A**), Hs-578T (**B**), and TCHBC5 (**C**) cells, measured by CCK-8 assay. Cells were co-treated with 10 ng/mL IL-1β and 20 μM Sch B for 24 h. (**D**–**G**) Representative Western blot images (**D**) and protein expression levels of EMT markers of MDA-MB-231 (**E**), Hs-578T (**F**), and TCHBC5 (**G**) cells. Cells were co-treated with 10 ng/mL IL-1β and 20 μM Sch B for 48 h. Original images can be found in Supplementary Materials. (**H**–**J**) mRNA expression levels of EMT markers of MDA-MB-231 (**H**), Hs-578T (**I**), and TCHBC5 (**J**) cells. Cells were co-treated with 10 ng/mL IL-1β and 20 μM Sch B for 24 h. Protein expression level was relative to that of α-tubulin. Data points represent three independent experiments, and bar graphs are presented as mean ± SD. * *p* < 0.05, ** *p* < 0.01, and *** *p* < 0.001 compared to vehicle group; # *p* < 0.05, ## *p* < 0.01, and ### *p* < 0.001 compared to IL-1β group. Significance according to one-way ANOVA.

Finally, an attempt to apply IL-1β on TNBC cells revealed a significant increase in cell proliferation (Figure 6A–C) and EMT expression (Figure 6D–G), confirming the pro-carcinogenic role of IL-1β. To this end, we analyzed whether Sch B can hinder this IL-1β-induced TNBC growth and progression. Our results first showed that 20 μM Sch B, although not always statically significant, can inhibit IL-1β-induced TNBC proliferation (Figure 6A–C). Regarding mesenchymal markers, apart from N-cadherin expression in MDA-MB-231 and TCHBC5 cells, most of the IL-1β-induced increase in mesenchymal markers was inhibited by Sch B (Figure 6D–G). Combining results from the mRNA (Figure 6H–J) suggests that Sch B can inhibit TNBC progression by altering the effect of IL-1β on the TNBC cells. Altogether, our data indicated that Sch B treatment can suppress the growth and progression of TNBC cells through downregulating NLRP3-derived IL-1β activation.

## 4. Discussion

Clinically, TNBC treatment has been challenging due to the disease’s aggressiveness, poor prognosis, and propensity for recurrence [4]. Although much effort has focused on finding an alternative therapeutic drug with higher effectiveness, no drugs are yet available to cure TNBC. 

Sch B, an isolated compound from *Schisandra chinensis*, has recently shown promise in cancer therapy [10,12,16,17]. Here, we reported that Sch B could effectively suppress the growth and progression of TNBC cells through inhibition of EMT and inflammasome activation. 

Although a previous study reported that STAT3 overexpression can rescue Sch-B-induced apoptosis of TNBC cell lines [17], the exact mechanism has yet to be addressed. Here, we provided evidence that Sch B can, through inhibition of the NLRP3-derived IL-1β production, suppress TNBC growth and progression. In addition to many oncogenic signaling pathways, such as JAK/STAT or PI3K/mTOR [20], inflammasome activation has long been suggested to promote EMT, leading to tumor growth and progression [18]. The production of IL-1β by the cancer cell also led to a more pro-tumor microenvironment [18], and its expression has been suggested to accelerate tumor growth [21,22], including many breast cancer cases [23,24,25]. Therefore, therapeutic targeting of IL-1β may provide one realistic approach against TNBC. Although the effect of Sch B on IL-1β has seldomly been observed in cancer models, its inhibitory effect on nuclear factor kappa-light-chain-enhancer of activated B cells (NF-ĸB), the central mediator of the priming signal of NLRP3 inflammasome, has been investigated in lung cancer cell lines [26]. However, whether Sch B inhibition of NF-ĸB would also occur in TNBC cells would require further study. 

EMT is a process by which epithelial cells lose their polarity and transit into an invasive, mesenchymal phenotype. In our study, we found that inflammasome inhibition by Sch B can accompany EMT inhibition, reducing TNBC progression. Our results agree with a previous study indicating inhibition of inflammasome activation and IL-1β signaling can decrease breast cancer growth and metastasis [27]. Clinical trials have identified increased serum IL-1β levels and intratumoral IL-1β expression in breast cancer patients, positively correlated to tumor stage and progression [28,29,30]. The increase in IL-1β allows the establishment of an inflammatory tumor microenvironment (TME) [31] and induces EMT and EMT memory through activation of the EMT transcription factors Snail and Slug [32,33]. Therefore, the reduction of Snail by Sch B (Figure 2J, Figure 5D and Figure 6D) may result from the inhibition of IL-1β. Although our study revealed reduced expression of mesenchymal markers by Sch B, the expression of epithelial marker E-cadherin was not changed. Although the loss of E-cadherin has largely contributed to tumor growth and invasiveness in animal models [34], the change in E-cadherin expression may not always happen in in vitro models [35,36,37]. It has been shown that a change in E-cadherin expression may or may not occur during EMT induction. Even ectopic expression of E-cadherin in cancer cell lines failed to prevent the EMT process, suggesting E-cadherin may not always impede EMT [36]. Therefore, it may be necessary to further investigate the role of E-cadherin during EMT. 

Previously, Sch B treatment has been shown to induce apoptosis in osteosarcoma [38], cholangiocarcinoma [10], gallbladder cancer [39], and also in another study using TNBC cell lines [17]. The two main pathways of apoptosis are extrinsic and intrinsic; the former requires caspase-8 activation, and the latter involves the formation of apoptosome and caspase-9 activation [40]. Caspase-8 has been shown to contribute to IL-1β processing; it can either cleave IL-1β directly [41] or through activation of inflammasome [42]. Therefore, it is believed that induction of apoptosis may also activate the inflammasome. However, our data here contradict this hypothesis, as Sch B inhibits inflammasome activation in TNBC cells. Previous study have suggested that Sch B may induce apoptosis in TNBC cell lines, but the involvement of caspases has not been investigated [17]. Also, we cannot exclude the possibility that Sch B induction of TNBC apoptosis and inflammasome inactivation may actually involve different pathways. In addition, the difference in Sch B concentration used (5, 10, and 20 μM in this study and 50 and 100 μM in the previous study) may also provide an explanation for these results, as a low drug dose and high drug dose may induce very different responses. Therefore, future work may be needed to investigate the correlation between apoptosis and inflammasome in TNBC cells. 

In this study, we included the use of one patient-derived TNBC cell line. This TCHBC5 cell line originated from a patient with metaplastic carcinoma with mesenchymal differentiation and TNBC phenotype, which is the most aggressive breast malignancy with the worst prognosis [43]. The patient of TCHBC5 has had prior treatment failure with most of the chemotherapy drugs and pembrolizumab. Currently, treating metaplastic breast cancer is still challenging, as knowledge regarding its tumorigenesis is still inadequate [43,44]. Our results here suggested an extraordinary effect of Sch B as a treatment option for this aggressive breast cancer; and it provided us with the idea that Sch B may serve as an adjuvant to current treatment options, either by consumption as a nutrient or combined with chemotherapeutic drugs. Yet, it is still questionable whether the concentration can still be achieved when Sch B is consumed as a nutrient or through bloodstream transportation. 

TNBC is an immunogenic cancer [4,45], suggesting that cytokine expression predominantly affects progression. Here, we presented that TNBC cells can secret IL-1β, possibly through autocrine signaling, which may affect cell growth. This IL-1β-induced TNBC growth was arrested by Sch B. Sch B has been proposed to largely affect cytokine regulation in vitro and in vivo [46]; therefore, in addition to IL-1β, the effect of Sch B on other TNBC-expressed cytokines may warrant further investigation, as expression of other cytokines such as IL-4 or IL-10 has also been shown to affect tumor growth [47,48]. 

## 5. Conclusions

In conclusion, Sch B hinders inflammasome activation and IL-1β production, inhibiting TNBC growth and progression. This research may serve as a base for future application of Sch B in combating TNBC.

## Figures and Tables

**Table 1:** Clinical characteristics of patient TCHBC5.

	TCHBC5
Patient characteristics	
Age (years)	61
Menopausal status	Postmenopause
Tumor histology	
Histological grade	III
Histological subtype	MCMD
Tumor characteristics	
Tumor stage	IIIB
TNM stage	
Tumor size (size in cm)	T4 (10, with extension to skin)
LN metastasis (number)	N3 (5)
Distant metastasis	M0
Molecular subtype	
HER2 expression	0%
ER expression	0%
PR expression	0%
Ki-67 expression	60%
Patient management	
Surgery type	RTM + ALND
Hormonal therapy	−
Chemotherapy	+
Cell culture	
Cell morphology	Spindle
Receptor expression	
HER2 expression	Negative
ER expression	Negative
PR expression	Negative

MCMD, metaplastic carcinoma with mesenchymal differentiation; HER2, human epidermal growth factor receptor 2; ER, estrogen receptor; PR, progesterone receptor; RTM, right total mastectomy; ALND, axillary lymph node dissection.

## Data Availability

The data in this study are not openly available due to reasons of sensitivity and patients’ confidentiality; but are available from the corresponding author upon reasonable request.

## Supplementary Materials

The following supporting information can be downloaded at: https://www.mdpi.com/article/10.3390/biom14010074/s1. Figure S1. Schisandrin B (Sch B) does not alter the growth of MCF-7A cells. (A) Viability of MCF-7A cells, treated with different concentrations of Sch B for 24 or 48 h. (B) Number of cells counted after treatment of Sch B. The cells were trypsined every 48 h, counted, and re-plated into a new 60 mm^3^ dish. Data points represent three independent experiments, and bar graphs are presented as mean ± SD. Figure S2. The effect of Schisandrin B (Sch B) on GSDMD levels of different TNBC cells. (A–C) MDA-MB-231 (A), Hs-578T cells (B), and TCHBC5 (C) cells were treated with Sch B for 48 h and extracted for GSDMD measurement. Data points represent three independent experiments, and bar graphs are presented as mean ± SD. * *p* < 0.05 and ** *p* < 0.01 compared to vehicle group. Significance according to one-way ANOVA. Table S1. Primer pairs used in this study. Table S2. Primary antibodies used in this study.
