# Supplementary figures and images for: The Use of Schisandrin B to Combat Triple-Negative Breast Cancers by Inhibiting NLRP3-Induced Interleukin-1β Production
Source: Biomolecules. 2024 Jan 5;14(1):74. doi: 10.3390/biom14010074 (PMC10813220; doi:10.3390/biom14010074)

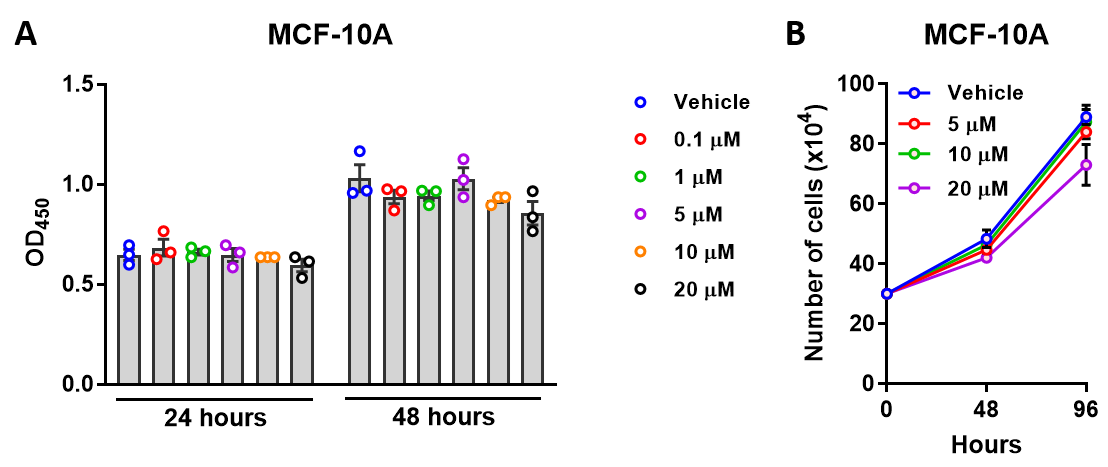

Supplement: Supplementary file 1 [file biomolecules-14-00074-s001.zip › biomolecules-2699189-Figure S1.png]

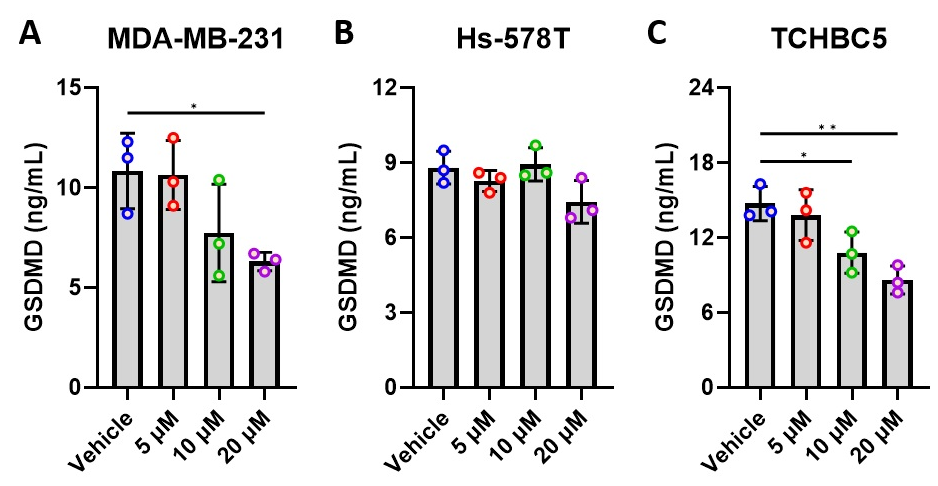

Supplement: Supplementary file 1 [file biomolecules-14-00074-s001.zip › biomolecules-2699189-Figure S2.png]
